# The Session of the General Medical Council

**Published:** 1897-06-05

**Authors:** 


### THE SESSION OF THE GENERAL MEDICAL COUNCIL.

The General Medical Council assembled for its sixty-second session on May 25th, and rose on the 31st. Sir Richard Quain, the President, with touching and courageous devotion to duty, although in his eighty-first year, rose from a sick bed to discharge the functions of his office, delivered the usual opening address on the business to be done, and presided daily, often for the whole and always for a considerable part of the sitting.

A special interest was given to the meeting by the appearance of two new members, Dr. Rentoul and Mr. Brown, as "direct representatives" of the medical profession in England. The retiring direct representatives, who did not seek re-election, were Sir Walter Foster, M.P., and Mr. Wheelhouse (of Leeds), both of them gentlemen belonging to the medical teacher class, and both possessing the kind of knowledge which is essential to the educational work of the Council, in which they were highly valued. At the last election, however, in consequence of an erroneous belief that the powers of the Council might in some way be exerted for the benefit of general practitioners, majorities of the fraction more than half of the profession which took the trouble to vote at all were cast for candidates who, by themselves or by their friends, had been active in giving currency to this delusion; and representatives were returned who were deeply pledged to the accomplishment of many things which, so far from being able to accomplish them, it would be impossible for the Council even to attempt. Some curiosity was therefore felt as to how the new representatives would adjust themselves to an environment which, in reality, is extremely different from any that may be supposed to have existed in their own imaginations, or, at least, in the imaginations of their supporters.

After the President's address had been delivered and acknowledged, and after a committee to arrange the order of business had been appointed, the first matter brought forward was the presentation of a loyal address to Her Majesty the Queen, on the approaching completion of the sixtieth anniversary of her reign. A committee was appointed to prepare such an address, and, on a subsequent day, it was duly signed by the members for transmission to the Home Office.

The Council then considered the direction of the Privy Council that they should appoint assistant examiners in surgery for the Apothecaries' Hall of Ireland, so as to enable that body to confer a complete registrable qualification. This subject also was referred to a committee; and, upon its report on a subsequent day, Mr. Chance, of Dublin, and Mr. Thomson, of Edinburgh, were duly appointed for a period of two years.

A letter was read from the Privy Council requesting the opinion of the Medical Council on the provisions of the Bill for the Registration of Midwives, now before Parliament; but, as it was manifest that the Bill, while likely to be re-introduced next session, could not possibly be proceeded with in the present one, it was determined to refer the question to a committee of the Council, and to consider their report at the meeting in November. Dr. Rentoul, who has greatly interested himself in the question, was placed upon the committee.

A highly-important penal case came under consideration. Dr. Robert Alexander Douglas Lithgow was charged by the Medical Defence Union with having "covered" an unregistered person, Dr. William Maunsell Collins, whose name had been erased from the Register in consequence of his having been convicted of forgery; and also with having covered an unqualified female practitioner, Mrs. Maitland King, who conducts, or has conducted, an establishment for the treatment of patients by electricity and massage. The case had for some time been under the notice of the Council, but was not previously thought to be ripe for consideration; and the result would probably now have been very uncertain had not Dr. Lithgow's counsel, Sir Frank Lockwood, Q.C., and Mr. Lush, thought proper to tender him as a witness. In this capacity he made admissions which were very damaging to his defence; and the Council ultimately found that he had been guilty of infamous conduct in a professional respect, and ordered his name to be erased from the Medical Register. In another case of "covering" an unqualified practitioner, the defendant was given time in which to abandon the practice complained of; and, in a third, in which the covering had been abandoned under pressure from the Council, the proceedings were allowed to drop. The name of Mr. Costelloe, which was erased from the Register in November, 1894, was restored on the recommendation of the Executive Committee.

Draft proofs of the new Pharmacopœia were submitted by the committee charged with its production; and it was announced that the complete work would, in all probability, be ready for issue by the November session of the Council.

Mr. Millar, who had been Registrar of the Council for more than 20 years, having been compelled to resign on account of ill-health, Mr. Allen, the former Assistant Registrar, was appointed to be his successor, and certain changes were made in the tenure and duties of the office.

Consideration was given to the question of the disciplinary powers of the various licensing bodies, some of which do, while others do not, possess the power of withdrawing qualifications from convicted or otherwise unworthy persons; and the subject was referred to a committee for further inquiry and report.

The Public Health Committee reported in strong terms on the question to which we referred last week, in relation to the Bill proposing to give power to Scottish local authorities to take proceedings for the abatement of nuisances on the certificate of a possibly ignorant "sanitary inspector," and the report was directed to be forwarded to the Secretary for Scotland and to the Lord Advocate. A memorial and petition, of which we elsewhere make mention, were prepared for the Government and for Parliament, seeking to remedy the diversion from the Medical Council of penalties inflicted for violations of the Medical Acts.

Dr. Rentoul, as "direct representative" and champion of the medical profession, presented a petition from Mr. R. B. Anderson, praying, among other things, that the Council would vote money in aid of litigation in which he was or intended to be engaged; but the Council being warned by its legal advisers that the proposed course of action would be *ultra vires* and absolutely illegal, declined to receive the petition. The case had been considered in a former session, and with practically the same result. Mr. Anderson appears to have suffered injustice, but it is not within the power of the Council to assist him in obtaining a remedy.

Mr. Brown was not fortunate enough to be in possession of any particular grievance. He spoke early and spoke often; not only more, and more frequently than any other member of Council, but probably about as much as all the others put together. He proved himself to be a past master of the art of talking a great deal while saying very little; and none of his speeches, most of which seemed to be made under the influence of erroneous impressions about simple matters of fact, were of a character to require careful record. They were chiefly meritorious in the direction of affording rest to the reporters.

